# Nasal high flow in management of children with status asthmaticus: a retrospective observational study

**DOI:** 10.1186/s13613-017-0278-1

**Published:** 2017-05-22

**Authors:** Florent Baudin, Alexandra Buisson, Blandine Vanel, Bruno Massenavette, Robin Pouyau, Etienne Javouhey

**Affiliations:** 1 Réanimation pédiatrique, Hôpital Femme Mère Enfant, Hospices Civils de Lyon, 69500 Bron, France; 2 UMR T_9405, UMRESTTE, Ifsttar, Université Claude Bernard Lyon1, Univ Lyon, 69373 Lyon, France

**Keywords:** Asthma, Children, High-flow nasal cannula, Non-invasive ventilation, Paediatric intensive care unit

## Abstract

**Background:**

Asthma is the most common obstructive airway disease in children and adults. Nasal high flow (NHF) is a recent device that is now used as a primary support for respiratory distress. Several studies have reported use of NHF as a respiratory support in status asthmaticus; however, there are no data to recommend such practice. We therefore conducted this preliminary study to evaluate NHF therapy for children with status asthmaticus admitted to our PICU in order to prepare a multicentre randomized controlled study.

**Results:**

Between November 2009 and January 2014, 73 patients with status asthmaticus were admitted to the PICU, of whom 39 (53%) were treated with NHF and among these 10 (26%) presented severe acidosis at admission (pH < 7.30). Thirty-four less severe children (41%) were treated with standard oxygen. For one child (2.6%) NHF failed and was then switched to non-invasive ventilation. NHF was discontinued in another patient because of the occurrence of pneumothorax after 31 h with NHF; the patient was then switched to standard oxygen therapy. Mean ± SD heart rate (165 ± 21 vs. 141 ± 25/min, *p* < 0.01) and respiratory rate (40 ± 13 vs. 31 ± 8/min, *p* < 0.01) decreased significantly, and blood gas improved in the first 24 h. In the subgroup of patients with acidosis, median [IQR] pH increased significantly between hour 0 and 2 (7.25 [7.21–7.26] vs. 7.30 [7.27–7.33], *p* = 0.009) and median [IQR] pCO_2_ decreased significantly (7.27 kPa [6.84–7.91 vs. 5.85 kPa [5.56–6.11], *p* = 0.007). No patient was intubated.

**Conclusion:**

This retrospective study showed the feasibility and safety of NHF in children with severe asthma. Blood gas and clinical parameters were significantly improved during the first 24 h. NHF failed in only two patients, and none required invasive ventilation.

## Background

Asthma is the most common obstructive airway disease in children and adults. Approximately 334 million people around the world and 2.5 million people in France suffer from asthma [1], a third of whom are children [1, 2], and the prevalence of asthma in this subpopulation has increased in recent decades [2]. Supplemental oxygen is commonly administered to children with an asthma exacerbation in the emergency department or intensive care unit in association with beta 2 agonist nebulization [3–5]. Non-invasive ventilation (NIV) may be used as respiratory support in children with status asthmaticus in case of standard treatment failure [6–9]. However, the level of evidence of its efficacy remains low according to the grade system of evidence quality [10].

Nasal high flow (NHF) is a recent device, now used as a primary support for respiratory distress in paediatric and adult intensive care units and in emergency departments [11–16]. It is increasingly used because it is well tolerated [11, 12, 17, 18] especially in infants with bronchiolitis [11, 17, 18]. NHF delivers humidified and heated gas at a rate greater than inspiratory flow [14, 19]. It reduces anatomical dead space by flushing the nasopharyngeal cavity and may improve CO_2_ clearance. It also provides a certain level of positive end-expiratory pressure (PEEP), between 2 and 7 cm H_2_O, depending on the flow rate used [14, 19–22] that may reduce resistance. In children with status asthmaticus, external PEEP may decrease work of breathing [23] based on the “waterfalls” principle published by Tobin and Lodato [24]. HFNC may also reduce the metabolic cost of breathing by supplying adequately warmed and humidified gas. Similarly, in infants with severe bronchiolitis, Milesi et al. demonstrated that HFNC significantly reduced work of breathing, respiratory rate, and Ti/Ttot ratio [25]. By increasing the expiratory time, HFNC may decrease dynamic hyperinflation in patients with obstructive lung disease and break the vicious circle.

There are, however, very few data reported NHF as a primary respiratory support for status asthmaticus, even though some studies have reported its use in the emergency department or intensive care unit in children [11, 12, 15, 16, 26, 27] as in adult patients [28, 29]. Over the previous five years NHF has been commonly used for children admitted to our PICU for acute respiratory failure (ARF) including patients with lower airway obstruction (bronchiolitis or asthma). We therefore conducted this preliminary study to evaluate NHF therapy for children admitted to our PICU with status asthmaticus in order to prepare a multicentre randomized controlled study.

## Methods

### Study design

We conducted a retrospective observational study in a 23-bed PICU of a tertiary university hospital (Hôpital Femme Mère Enfant, Lyon University Hospital, France). Children aged between 1 and 18 years, without severe comorbidities, admitted between November 2009 and January 2014 to the PICU, and with a diagnosis of status asthmaticus were included. The study was approved by our institutional review board and a waiver of consent given (CPP Sud-Est II N°00009118—2016-08).

### Population

Patients were identified in the French hospital information system (PMSI) and the PICU database by using the primary diagnosis of status asthmaticus (International Classification of Diseases—ICD 10 code J46) or ARF associated with asthma (ICD 10 J96.0/J45). Based on the local protocol and French recommendations [5], children were admitted to the PICU after at least 1 h in the emergency department during which they did not response to standard therapy, based on at least three successive beta agonist nebulizations, supplemental oxygen, and oral or intravenous corticosteroids at 2 mg/kg.

In PICU, respiratory support (oxygen, HFNC, NIV, or invasive ventilation—IV) and additional therapy (intravenous salbutamol, magnesium sulphate) were left to the physician’s judgment. Patients with severe comorbidities were excluded: cardiopulmonary disease, neuromuscular or metabolic disease, restrictive or chronic respiratory disease (pulmonary fibrosis, cystic fibrosis, bronchodysplasia), ENT disease (laryngo- or tracheo- or broncho-malacia) or children with tracheotomy. For NHF, Optiflow RT330 (Fisher & Paykel Healthcare, Auckland, New Zealand) circuit and nasal prong adapted to the age and the size of the nose were used. The nebulizer system (Aerogen, Inc., Mountain View, CA, USA) was inserted upstream from the electrically heated humidifier [30–32].

### Data and outcome

Data were retrospectively collected using the electronic medical record IntelliSpace Critical Care and Anesthesia (Philips Healthcare, Suresnes, France). A patient was attributed to only one group (NHF or standard oxygen), and in case of multiple stays during the period, only the first one was analysed. The primary outcome was defined as failure of the NHF therapy and described as a proportion of all children with asthma having received NHF therapy. The secondary outcome was the change of clinical parameters (respiratory rate, heart rate, SpO_2_/FiO_2_ ratio) from NHF initiation to 6, 12, 24, and 48 h later, as well as blood gas parameters in children treated with NHF.

Baseline characteristics of the population (age, weight, comorbidity, history of asthma) were collected at admission and compared to those of the standard oxygen group. Data on the medication used before and during PICU stay, and the duration of NHF use and of supplemental oxygen therapy, and length of PICU stay were also collected. Safety of HFNC treatment was assessed by the number of patients with air-leak complications and by the tolerance of the system according to nurse reports. A subgroup analysis of children with severe acidosis treated with NHF was also performed.

### Statistical analysis

Qualitative variables are reported as numbers and percentages, and quantitative variables are reported as mean ± standard deviation (SD) or confidence intervals, or as median with interquartile range [IQR], when appropriate. Chi-square test or Fisher’s exact test for qualitative variables and Mann–Whitney *U* test for nonparametric independent sample were used to compare the data between NHF and standard oxygen groups, when appropriate. Repeated-measures analysis of variance (ANOVA) was used to compare clinical variables over time. The assumption of sphericity was tested using Mauchly’s test of sphericity; if sphericity was violated epsilon (*ε*) was calculated according to Greenhouse and Geisser and used to correct the one-way repeated-measures ANOVA [33]. Post hoc analysis was performed with a Bonferroni adjustment. Wilcoxon signed-rank test was used for nonparametric paired samples. Differences were considered statistically significant at *p* < 0.05. Statistical analysis was performed using SPSS Statistics (V22, IBM, Armonk, NY, US).

## Results

**Fig. 1:**
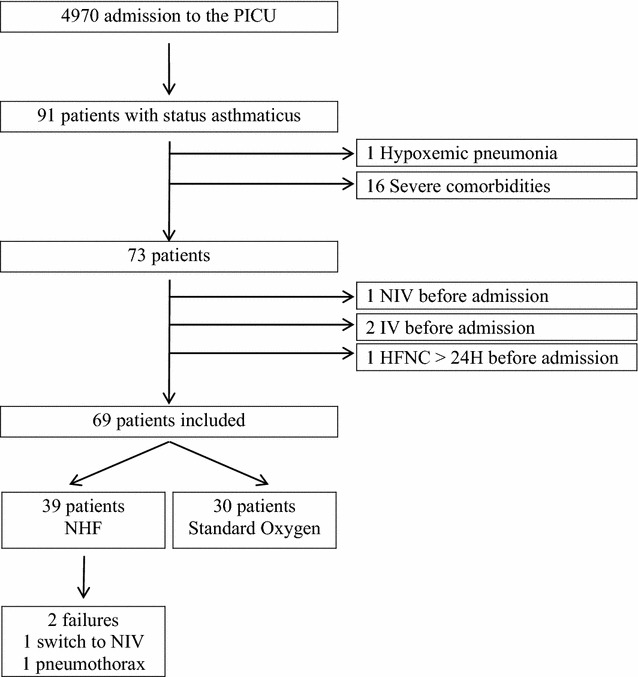
Patient flow chart. *PICU* paediatric intensive care unit, *NHF* nasal high flow, *NIV* non-invasive ventilation, *IV* invasive ventilation

**Fig. 2:**
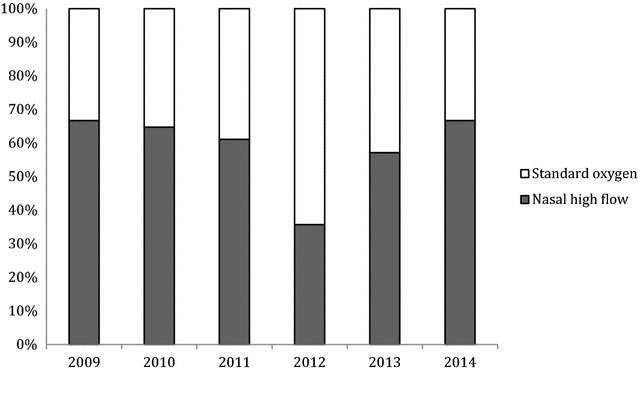
Proportion of children treated by nasal high flow and standard oxygen from 2009 to 2014 (*p* = 0.66 with Fisher’s exact test). *NHF* nasal high flow

Between November 2009 and January 2014, 91 children with diagnosis of status asthmaticus were admitted in our PICU. Sixteen children were excluded because of the presence of severe comorbidities and one because the primary diagnosis was hypoxemic pneumonia. Among the 73 children admitted for status asthmaticus, 39 (53%) were treated with NHF and 30 (41%) received only standard supplemental oxygen therapy (16 with non-rebreathing mask and 14 with standard nasal cannula, Fig. 1). The proportion of children treated by standard oxygen and NHF in each year of the study period was similar (*p* = 0.66) (Fig. 2). A further two children were intubated before admission to PICU (for transport): one was treated with NIV, and one was admitted in the PICU more than 24 h after starting NHF in an intermediate care unit outside of the university hospital (Fig. 1). The median [IQR] age of children treated with NHF was 3.6 years [1.6–5.6], which was similar to that of children treated with standard oxygen (3.6 [2.2–6.7]; *p* = 0.72). All children in the two groups received nebulized salbutamol and corticosteroids (intravenous corticosteroid for 79% in NHF and 63% in standard oxygen group). Continuous intravenous salbutamol was used in 13 children (33%) in the NHF group and in 5 (17%); *p* = 0.12. Magnesium sulphate was more often used in the NHF group (59%) than in standard oxygen group (27%, *p* = 0.007; Table 1).

**Table 1:** Baseline characteristics of children treated with nasal high flow and with standard oxygen therapy for status asthmaticus

	NHF *n* = 39	Standard oxygen *n* = 30	p*
Age (years), median [IQR]	3.6 [1.6–5.6]	3.6 [2.2–6.7]	0.72
Male/female ratio	20/19	21/9	0.11
Weight (kg), median [IQR]	15 [11–24]	15 [13–23]	0.64
PIM2 at admission, median [IQR]	1.5 [1.15–3.3]	1 [0.3–1.37]	<0.001
History of asthma or >2 bronchiolitis, *n* (%)	31 (80)	23 (77)	0.79
Previous admission for asthma, *n* (%)	19 (48)	11 (37)	0.31
In PICU, *n* (%)	4 (10)	2 (6)	0.66
Long-term control medicine, *n* (%)	17 (44)	14 (47)	0.80
Clinical parameters at admission, median [IQR]			
Respiratory rate (/min)	35 [31–47]	35 [30–43]	0.47
Heart rate (/min)	164 [154–185]	168 [153–180]	0.89
SpO_2_ (%)	97 [95–98]	98 [97–100]	0.04
SpO_2_/FiO_2_	216 [175–303]	NA	
Venous blood gas at admission, median [IQR]			
pH	7.35 [7.28–7.39]	7.36 [7.34–7.39]	0.27
pCO_2_ (kPa)	5.6 [4.7–7.7]	4.9 [4.4–5.6]	0.02
Bicarbonates (mmol/L)	22 [20–24]	20 [20–23]	0.35
Acidosis (pH < 7.30), *n* (%)	10 (26%)	2 (7%)	0.04
Associated medication, *n* (%)			
Salbutamol—nebulized	39 (100%)	30 (100%)	1.0
Corticosteroids—intravenous^a^	31 (79%)	19 (63%)	0.14
Salbutamol—intravenous	13 (33%)	5 (17%)	0.12
Magnesium sulphate	23 (59%)	8 (27%)	0.007
PICU LOS (days), median [IQR]	3 [2.5–5]	1.5 [1, 2]	<0.001

*LOS* length of stay, *PIM* Paediatric Index of Mortality, *PICU* paediatric intensive care unit, *NHF* nasal high flow

* Statistical analysis with Chi-square test for qualitative variables or Mann–Whitney *U* test for nonparametric independent sample

^a^All other children received oral corticosteroids

The median [IQR] flow of NHF was initially set at 0.9 L/kg/min [0.75–1] with a median [IQR] FiO_2_ of 45% [31–55] (Table 2). NHF failed in only two children. One child required NIV because of worsening blood gas parameters in the first 24 h. NHF was discontinued in another patient because of the occurrence of pneumothorax. The pneumothorax occurred after 31 h with NHF (X-ray at admission without pneumothorax) and requiring chest tube for 24 h. The maximum NHF was 1 L/kg/min. NHF was discontinued and standard oxygen therapy was administered at 0.5 L/min for 22 h. No patient was intubated. The median [IQR] length of NHF treatment was 28 h [21–47], and the median PICU length of stay was 3 days [2.5–5].

**Table 2:** Nasal high flow (NHF) parameters of 39 children treated for status asthmaticus

	*n* = 39
NHF settings, median [IQR]	
Initial FiO_2_ (%)	45 [31–55]
Initial flow (L/kg/min)	0.9 [0.75–1]
Maximum flow (L/kg/min)	1.0 [0.8–1.1]
Length of NHF (h), median [IQR]	28 [21–47]
NHF failure, *n* (%)	2 (6)

*PICU* paediatric intensive care unit, *NHF* nasal high flow

**Fig. 3:**
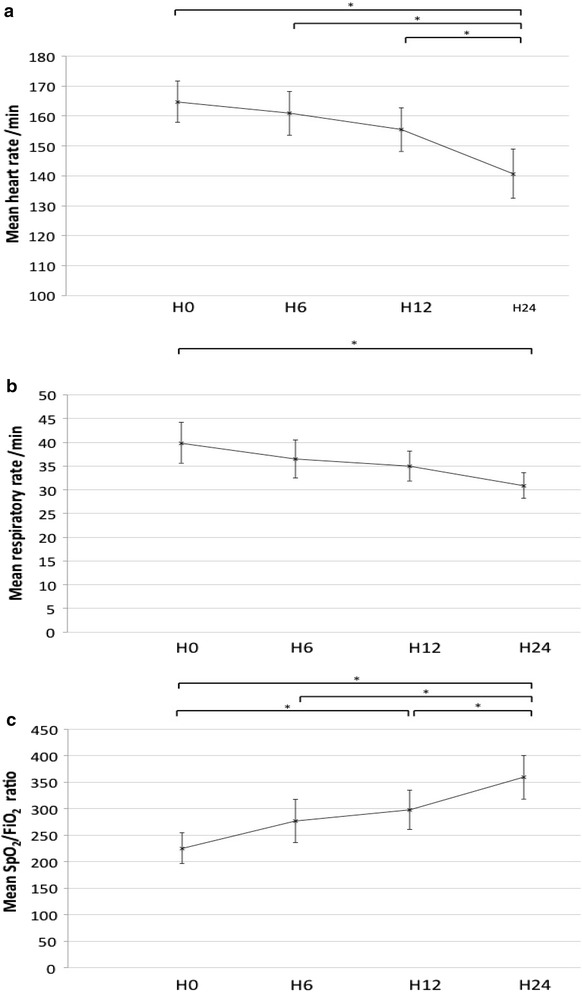
Change of heart rate (**a**), respiratory rate (**b**), and SpO_2_/FiO_2_ ratio (**c**) during the first 24 h in 38 children with status asthmaticus treated by nasal high flow. Heart rate, respiratory rate, and SpO_2_/FiO_2_ ratio significantly change over time according to the repeated-measures analysis of variance (ANOVA). *Significant difference with pairwise post hoc analysis (*p* < 0.01). *Bars* indicate 95% confidence intervals. *H* hours

Change of heart rate (HR) and respiratory rate (RR) during the first 24 h are presented in Fig. 3. The assumption of sphericity was violated for HR (*p* = 0.016), and a Greenhouse–Geisser correction was applied (*ε* = 0.82). HR decreased significantly over time *F*(2.47, 91.41) = 22.77, *p* < 0.001, partial η2 = 0.38, as did RR *F*(3, 111) = 8.65, *p* = 0.001, partial η2 = 0.19. Pairwise post hoc analysis found that mean ± SD HR and RR were significantly lower at hour 24 (141 ± 25 per min and 31 ± 8 per min, respectively) than at hour 0 (165 ± 21 per min, *p* < 0.01 and 40 ± 13 per min, *p* < 0.01). HR was also lower at hour 24 (141 ± 25 per min) than at hour 12 (155 ± 22 per min, *p* < 0.01) and at hour 6 (161 ± 22 per min, *p* < 0.01). For SpO_2_/FiO_2_ ratio the assumption of sphericity was also violated (*p* < 0.01) and a correction was applied (*ε* = 0.33). SpO_2_/FiO_2_ ratio changed significantly over time *F*(2.1, 67.0) = 19.7, *p* < 0.001, partial η2 = 0.38. SpO_2_/FiO_2_ ratio was higher at hour 24 (359 ± 116) than at hour 12 (298 ± 104, *p* < 0.01), at hour 6 (277 ± 116, *p* < 0.01), and at hour 0 (225 ± 81, *p* < 0.01); it was also higher at hour 12 (298 ± 104) than at hour 0 (225 ± 81, *p* < 0.01; Fig. 3). Blood gas (pH and PCO_2_) improved in the first 24 h for children treated with NHF (Table 3). Blood gas parameters were available at day 1 for only half of patients treated with standard oxygen (*n* = 15); the median [IQR] pH was 7.41 [7.38–7.42]; and pCO2 was 4.6 kPa [4.2–4.7].

**Table 3:** Change of blood gas parameters between hour 0 and 24 in children treated with nasal high flow for status asthmaticus

	Hour 0 *n* = 39	Hour 24 *n* = 37^a^	*p*
Venous blood gas, median [IQR]			
pH	7.35 [7.28–7.39]	7.42 [7.39–7.44]	*p* < 0.001
pCO_2_ (kPa)	5.6 [4.7–7.7]	4.3 [4.0–4.8]	*p* < 0.001
Bicarbonates (mmol/L)	22 [20–24]	21 [19–22]	*p* = 0.16

^a^Nasal high flow failed for one patient during the first 24 h, and one patient had no blood gas at day 1

**Fig. 4:**
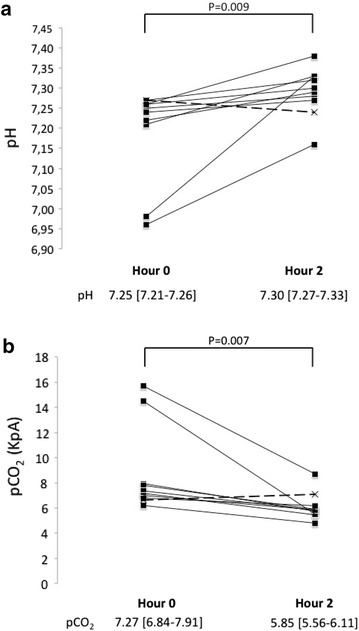
Change of pH (**a**) and pCO_2_ (**b**) at admission and at hour 2 in ten children with severe acidosis treated with nasal high flow for status asthmaticus, including one child (*discontinuous line*) who failed to HFNC

Ten patients treated with NHF (6 boys and 4 girls), who had a median [IQR] age of 3.7 years [2.1–4.4], had at severe acidosis at admission (pH < 7.30). In this subgroup, median [IQR] pH increased significantly between hour 0 (7.25 [7.21–7.26]) and hour 2 (7.30 [7.27–7.33], *p* = 0.009), and pCO_2_ decreased significantly (hour 0: 7.27 kPa [6.84–7.91], hour 2: 5.85 [5.56–6.11], *p* = 0.007; Fig. 4). In the patient who failed in the first 24 h (discontinuous line in Fig. 4), blood gases worsened from hour 0 to hour 2; the child was switched to non-invasive ventilation with success (Fig. 1).

## Discussion

The present study is the largest report to have evaluated the use of NHF as a primary respiratory support for severe status asthmaticus. It showed the feasibility and the safety of management of children with status asthmaticus with NHF; NHF failed in only one patient, and blood gas and clinical parameters were significantly improved during the first 24 h.

During the study period, 39 children were treated with NHF and 30 with standard oxygen. The demographic data were similar in terms of age, weight, and medical history. However, NHF was used according to the physician’s judgment (as was the use of additional therapy) and those who received standard oxygen seemed to be less severe at admission (lower PIM2 score, lower pCO_2_ values, and less frequently had acidosis) although clinical parameters (heart and respiratory rate) were similar. Another marker of severity is the administration of magnesium sulphate that, in our PICU, is recommended as a second-line therapy before the use of intravenous salbutamol and this was used twice less frequently in the standard oxygen group. Furthermore, the length of PICU stay was also longer in the NHF group, but is of note that both NHF had to be discontinued and nebulization to be scheduled less than every 3 h for patients to be discharged. These differences preclude any strong conclusions as to the superiority of one technique over the other, which is coherent with the nature of this preliminary retrospective study. It is of note that no patient was intubated (in either group) and only one required NIV. Furthermore, clinical parameters (heart rate and respiratory rate) improved over time with NHF as did blood gas values, even in children with severe acidosis. These results are strengthened by the efforts made to reduce bias related to patient identification and missing data that affect many other retrospective studies. This was limited herein by the use of status asthmaticus and ARF associated with asthma diagnosis codes, and electronic medical records with automatic importation of clinical and biological parameters every 5 min. However, improvement of the physiological parameters may also be due to the normal change over time and more robust conclusions will be made from the results of the multicentre randomized controlled trial that will be implemented later this year.

The place of NHF in the management of ARF is controversial. Several physiological studies have supported that NIV relieves better work of breathing than continuous positive airway pressure [34, 35] and therefore that it is better than NHF [18]. However, the most recent studies in adults suggest either superiority of NHF over conventional oxygen [36], or equivalence [37] and even superiority over NIV [38]. Pulmonary function may be affected by emotion and stress [39, 40], and tolerance to NHF is better than NIV, both in adults [41, 42] and in children [18, 43], and may explain in part the benefit of NHF. It was not possible to assess comfort of children retrospectively. After analysis of nurse report forms, no notable discomfort was reported, and in particular no skin lesions. Clinical improvement observed with NHF in the present study was similar to that previously reported with NIV in children [7], and no patient was intubated. However, although the use of NIV for status asthmaticus in children [6, 7, 9, 44, 45] is common, the level of evidence remains limited [10]. Furthermore, in adults, the Cochrane review published in 2012 found that NIV did not provide additional benefit to medical treatment [46]. At this time, the use of NHF in the most severe asthmatic patients may not be recommended as current guidelines indicate that intubation should never be delayed [47], even though the benefit of NHF in this subgroup was particularly demonstrative and rapid herein. On the other hand, using NHF to treat all children with mild asthma would lead to increase costs but not the benefits. Therefore, it would be of great interest to define the population who would most benefit from NHF, for which the preschool respiratory assessment measure (PRAM) [48] could be of interest. In our PICU, NHF is currently used as the primary respiratory support for children with moderate-to-severe asthma, defined by an acidosis (pH < 7.35) or a PRAM score >7 after optimal care in the emergency department. For severe patients, a senior physician systematically evaluates children at 1 h and blood gases are measured after 2 h of use to ensure an early detection of patients who do not improve.

NHF allows the delivery of nebulized drugs (i.e. beta agonists) continuously and without changing the interface [30–32, 49, 50] as during NIV. Recent studies suggest greater efficacy of vibrating mesh nebulizers over jet nebulizers [30, 31]. The former was used in association with NHF, and a jet nebulizer was used for children treated with standard oxygen, which further complicates interpretation of the results. More generally, delivery of beta agonist with NHF is heterogeneous and depends on several aspects. According to the manufacturer recommendations and recent studies [30–32], the nebulization system was placed upstream from the active heated humidifier that seems to provide better effectiveness. The gas flow rate is probably the main parameter to take into account the delivery of nebulization drugs. A recent study showed that in infants and toddlers, increasing the flow rate by fourfold decreases tenfold the proportion of lung deposition [32]. For asthma patients, it is necessary to weigh the benefit/risk ratio of a higher flow with higher respiratory support but probably with a decrease of drug delivery. In the present study, the median flow rate was 0.9 L/kg/min [0.75–1] that remains relatively low for paediatric patient [14]. A lower flow rate may participate to a better nebulization drug delivery and a better tolerance in children, older than patient with bronchiolitis.

In conclusion, this study shows that NHF is feasible in children with status asthmaticus, may improve physiological parameters, and prevent the use of subsequent therapeutic steps. Based on these results, a multicentre randomized controlled study will start later this year to evaluate whether early management with NHF may prevent failure in comparison with conventional oxygen (and therefore escalation to NIV or IV) in patients with moderate-to-severe asthma defined as an acidosis (pH < 7.35) or a PRAM score >7 after optimal care in the emergency department.

## Abbreviations

ANOVA: analysis of variance
ARF: acute respiratory failure
NHF: nasal high flow
HR: heart rate
ICD: International Classification of Diseases
IQR: interquartile range
IV: invasive ventilation
NIV: non-invasive ventilation
PEEP: positive end-expiratory pressure
PICU: paediatric intensive care unit
RR: respiratory rate
SD: standard deviation
